# Color Blindness

**Published:** 1881-06

**Authors:** 


### COLOR BLINDNESS.

The following is an act which was passed by the Massachusetts Legislature, April 11, 1881, relative to the employment by railroad companies of persons affected with defective sight or color blindness:

Sect. 1. No railroad company shall employ or keep in its employment any person in a position which requires him to distinguish form or color signals, unless such person within two years next preceding has been examined for color blindness or other defective sight, by some competent person employed and paid by the railroad company, and has received a certificate that he is not disqualified for such position by color blindness or other defective sight. Every railroad company shall require such employe to be re-examined at least once within every two years, at the expense of the railroad company.

Sect. 2. A railroad company shall be liable to a fine of one hundred dollars for each violation of the preceding section.

Sect. 3. This act shall take effect on the first day of July next.

